# Case for diagnosis. Erythematous-violaceous reticulated plaques on the breasts^☆^

**DOI:** 10.1016/j.abd.2021.07.005

**Published:** 2022-02-10

**Authors:** Bruno de Castro e Souza, Esio Pessoa Caracas de Souza, Neusa Yuriko Sakai Valente, José Antonio Sanches

**Affiliations:** Department of Dermatology, Universidade de São Paulo, Hospital das Clínicas, Universidade de São Paulo, São Paulo, SP, Brazil

## Case report

**Figure 1:**
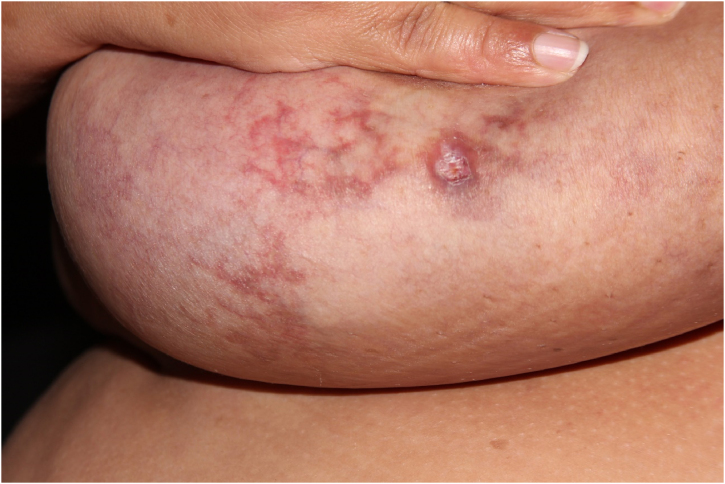
Large and pendulous breasts, with erythematous-violaceous reticulated plaques and some ulcerated areas.

A 46-year-old female patient came to the Dermatology outpatient clinic complaining of asymptomatic breast lesions for ten years. She reported a weight gain of approximately 50 kg during this period and reported being an active smoker with a tobacco load of 42 pack/year. Dermatological examination showed erythematous to violaceous reticulated lesions on both breasts. Some areas showed shallow ulcerations (Fig. 1).

**Figure 2:**
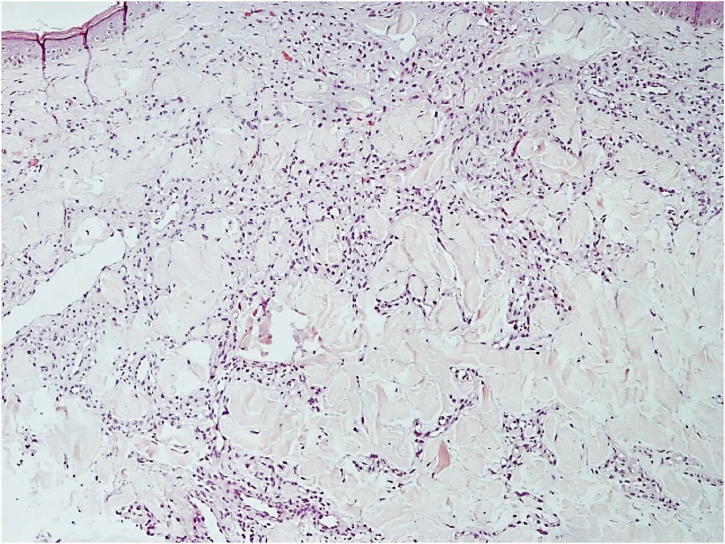
Proliferation of small vessels, more intense in the upper dermis, extending to the middle dermis (Hematoxylin & eosin, ×200).

**Figure 3:**
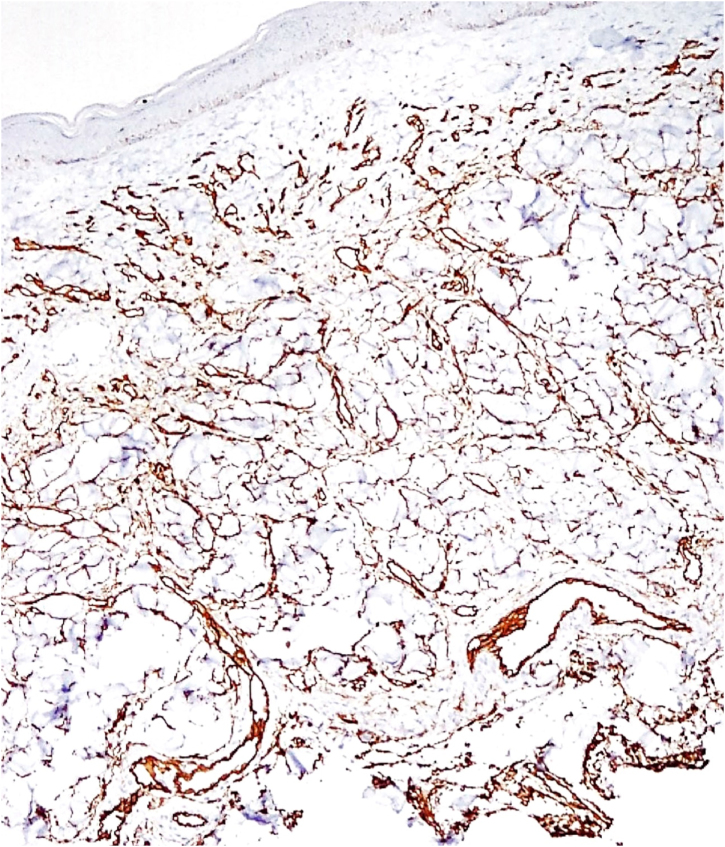
Immunohistochemistry enhancing the vascular endothelial proliferation with CD34.

Histopathology revealed a rectified epidermis and proliferation of small vessels, more intense in the upper dermis, but extending to the middle dermis without cell atypia (Fig. 2). The immunohistochemical analysis showed positivity for CD31 (vascular endothelium), CD34 (vascular endothelium and dermal dendrocytes) and negativity for D2-40 and HHV-8 (Fig. 3).

## What is your diagnosis?

a) Kaposi's Sarcoma
b) Low-grade angiosarcoma
c) Diffuse dermal angiomatosis of the breast
d) Calciphylaxis

## Discussion

**Figure 4:**
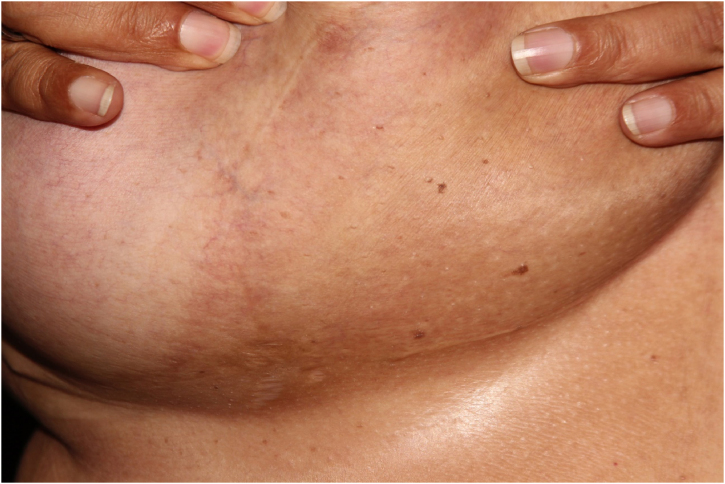
Complete resolution of lesions after loss of 20 kg.

Based on the clinical-pathological correlation, a diagnosis of diffuse dermal angiomatosis of the breast was made. Laboratory tests including ANF, lupus anticoagulant, anticardiolipin, anti-beta-2 glycoprotein, C and S proteins were normal. The CT angiography excluded venous or arterial thrombosis. The patient lost 20 kg in one year and there was complete regression without any medical or surgical treatment (Fig. 4). Despite medical advice, the patient did not stop smoking.

Diffuse dermal angiomatosis of the breast (DDAB) is a poorly described entity that affects exclusively middle-aged, obese women with macromastia and who are smokers.^1^, ^2^, ^3^ Clinically, the lesions range from reticulated erythematous patches to indurated plaques surrounded by opaque erythema. Histopathological examination demonstrates endothelial cell proliferation between collagen fibers. Small vascular lumens constituted by spindle-shaped endothelial cells with vacuolated cytoplasm are formed throughout the dermis. This characteristic can be better demonstrated by the immunohistochemical markers CD31 and CD34 (endothelial cell markers).^3^ The histological differential diagnoses include acroangiodermatitis, Kaposi's sarcoma, low-grade angiosarcoma, and reactive angioendotheliomatosis. The negativity for HHV-8 rules out the possibility of Kaposi's sarcoma.

The pathogenesis likely involves tissue ischemia leading to an increase in endothelial growth factors and neoangiogenesis.^4^ The cause of ischemia is variable and includes peripheral vascular disease, hypercoagulability, monoclonal gammopathy, Takayasu's arteritis, and calciphylaxis.^2^, ^3^, ^4^, ^5^, ^6^ Notably, this patient and almost all other reported cases had a history of smoking.^4^

The management of DDAB requires treatment of the underlying cause of tissue ischemia. The investigation to exclude the causes of ischemia is mandatory. Advice on weight loss, smoking cessation, and control of cardiovascular risk factors such as hypertension or hyperlipidemia are essential. Drug therapy includes the use of isotretinoin, anticoagulants, acetylsalicylic acid, pentoxifylline, and oral corticosteroids, but responses are variable.^2^, ^3^, ^4^, ^5^, ^6^, ^7^ Surgery is also an option, and there are some cases that showed resolution after reduction mammoplasty. It is noteworthy that, in some cases, such as the one presented in this report, weight loss alone may be enough to resolve the lesions without the need for medication or surgery.

## Financial support

None declared.

## Authors' contributions

Bruno de Castro e Souza: Drafting and editing of the manuscript; critical review of the literature.

Esio Pessoa Caracas de Souza: Collection, analysis, and interpretation of data; drafting and editing of the manuscript; critical review of the literature.

Neusa Yuriko Sakai Valente: Design and planning of the study; drafting and editing of the manuscript; critical review of the literature; intellectual participation in the propaedeutic and/or therapeutic conduct of the studied case.

José Antonio Sanches: Design and planning of the study; drafting and editing of manuscript; critical review of the literature; intellectual participation in the propaedeutic and/or therapeutic conduct of the studied case.

## Conflicts of interest

None declared.
